# Associations Between Intake of Fermented Dairy Products and Blood Lipid Concentrations Are Affected by Fat Content and Dairy Matrix – The Tromsø Study: Tromsø7

**DOI:** 10.3389/fnut.2021.773468

**Published:** 2021-11-22

**Authors:** Monika Lund Machlik, Laila Arnesdatter Hopstock, Tom Wilsgaard, Patrik Hansson

**Affiliations:** ^1^Department of Clinical Medicine, UiT the Arctic University of Norway, Tromsø, Norway; ^2^Department of Community Medicine, UiT the Arctic University of Norway, Tromsø, Norway

**Keywords:** fermented dairy products, yogurt, cheese, dairy matrix, blood lipids, LDL-Cholesterol, HDL-Cholesterol, triglycerides

## Abstract

**Introduction:** Dairy fat is rich in saturated fatty acids known to increase serum low-density lipoprotein cholesterol (LDL-C) concentration, an important risk factor for cardiovascular disease (CVD). However, intake of fermented dairy products has been associated with reduced CVD risk in observational studies. How intakes of different fermented dairy products are associated with blood lipid concentrations may provide a possible explanation for the suggested reduced CVD risk.

**Aim:** To examine the associations between different types of fermented dairy products, with various fat contents and dairy matrix structures, and blood lipid concentrations in a general population.

**Methods:** In 11,377 women and men aged between 40-99 participating in the population-based Tromsø Study 2015-2016, multivariable linear regression was used to examine associations between total intake of fermented dairy products, intake of yogurt (including regular-fat, low-fat, and semi-solid yogurt), cheese (including regular-fat and low-fat), and liquid fermented dairy, and serum concentrations of total cholesterol, LDL-C, high-density lipoprotein cholesterol (HDL-C), and triglycerides. Dietary data was collected using a validated food frequency questionnaire. Analyses were adjusted for potential confounding factors, and cheese intake analyses were stratified by self-reported use of cholesterol-lowering drugs.

**Results:** Cheese intake was positively associated with HDL-C [regression coefficient 0.02 mmol/l (95 % CI 0.01, 0.03)], and inversely associated with LDL-C [regression coefficient−0.03 mmol/l (95 % CI−0.04,−0.01)] and triglycerides [relative change −1.34 % (95 % CI: −2.29 %, −0.37 %)] per 25 g/day among non-users of cholesterol-lowering drugs, while no associations were found among users. Total intake of fermented dairy was inversely associated with triglycerides [relative change −1.11 % (95 % CI: −1.96 %, −0.24 %)] per 250 g/day, while no associations were found for yogurt intake. Intake of low-fat cheese was more favorably associated with blood lipids compared to regular-fat cheese, and semi-solid yogurt was inversely associated with LDL-C and triglycerides, while intake of liquid fermented dairy was not associated with any of the blood lipids.

**Conclusion:** This study highlights the importance of investigating specific types of dairy products separately, based on fat content and dairy matrix, when examining effects on blood lipid concentrations, and stratifying statistical models by use of cholesterol-lowering drugs when relevant.

## Introduction

Cardiovascular disease (CVD) is the main cause of death worldwide, accounting for about one third of the deaths globally in 2019 (1). The main underlying cause of most CVD is atherosclerosis, an inflammatory process involving retention of apolipoprotein B-containing remnant particles, i.e. low-density lipoprotein (LDL) particles and triglyceride-rich lipoprotein remnants, into the arterial wall (1–3). Thus, elevated concentrations of these lipoproteins are a major risk factor for atherosclerotic CVD (4, 5). In addition, low concentration of high-density lipoprotein cholesterol (HDL-C) is inversely associated with risk of atherosclerotic CVD, even though clinical trials have failed to find a reduced risk by increasing plasma HDL-C (5).

Dairy products are major contributors to intake of saturated fat, accounting for about 46% of the total intake of saturated fat in Norway (6) and 25–36% of the total intake of saturated fat in the UK (7, 8). About 70% of the fatty acids in dairy products are saturated fatty acids, and 11% are myristic acid (14:0) and 29% are palmitic acid (16:0) (9). Both these saturated fatty acids have been shown to increase the LDL-C concentration (10). Despite the high saturated fat content in dairy products, a recent review article including meta-analyses of cohort studies found no associations between total dairy product consumption (including regular-fat and low-fat dairy products, high compared with low consumption, and dose–response intake) and risk of CVD (11). The same review article conducted a meta-analysis of randomized controlled trials (RCTs) conducted between 2013–2018. The meta-analysis found that total dairy intake had no significant effect on total cholesterol or LDL-C concentrations (11). In addition, a meta-analysis of RCTs, including healthy adults randomized to increased dairy food intake for more than a month without other dietary interventions, found that neither regular-fat dairy nor low-fat dairy had a significant effect on LDL-C or HDL-C (12). Moreover, several meta-analyses of randomized controlled trials show that intake of fermented dairy products are inversely associated with total cholesterol and LDL-C concentrations (13–16). However, fermented dairy products is a heterogeneous food group consisting of a large number of products differing in both nutrient composition, such as fat content, and dairy matrix structure, indicating that specific fermented dairy products may have different effects on blood lipid concentrations (17–19). This is supported by postprandial studies showing different effects on triglycerides, HDL-C, and lipoprotein subclass concentrations between fermented dairy products that differ in dairy matrix structure (20–22). Thus, the aim of this study is to examine the associations between total intake of fermented dairy products, as well as intake of yogurt (including both low-fat and regular-fat), cheese (including both low-fat and regular-fat), semi-solid yogurt, and fermented liquid dairy, respectively, and blood lipid concentrations in a general population.

## Materials and Methods

### Sample and Data Collection

The Tromsø Study is a population-based cohort study conducted in Tromsø, Norway, which includes seven repeated surveys conducted since 1974 (23). Total birth cohorts and random samples have been invited to participate in the study, with an attendance of 65–85% (24, 25). The study population in this cross-sectional study consists of participants from the seventh survey, Tromsø7 (2015–2016). Data collection included general questionnaires and interviews, a food frequency questionnaire (FFQ), clinical examinations, and biological sampling. Participants with valid data on exposure variables, outcome variables and covariates in the regression analyses (derived from questionnaires, FFQ, and biological samples) were included in the study.

### Study Sample

**Figure 1:**
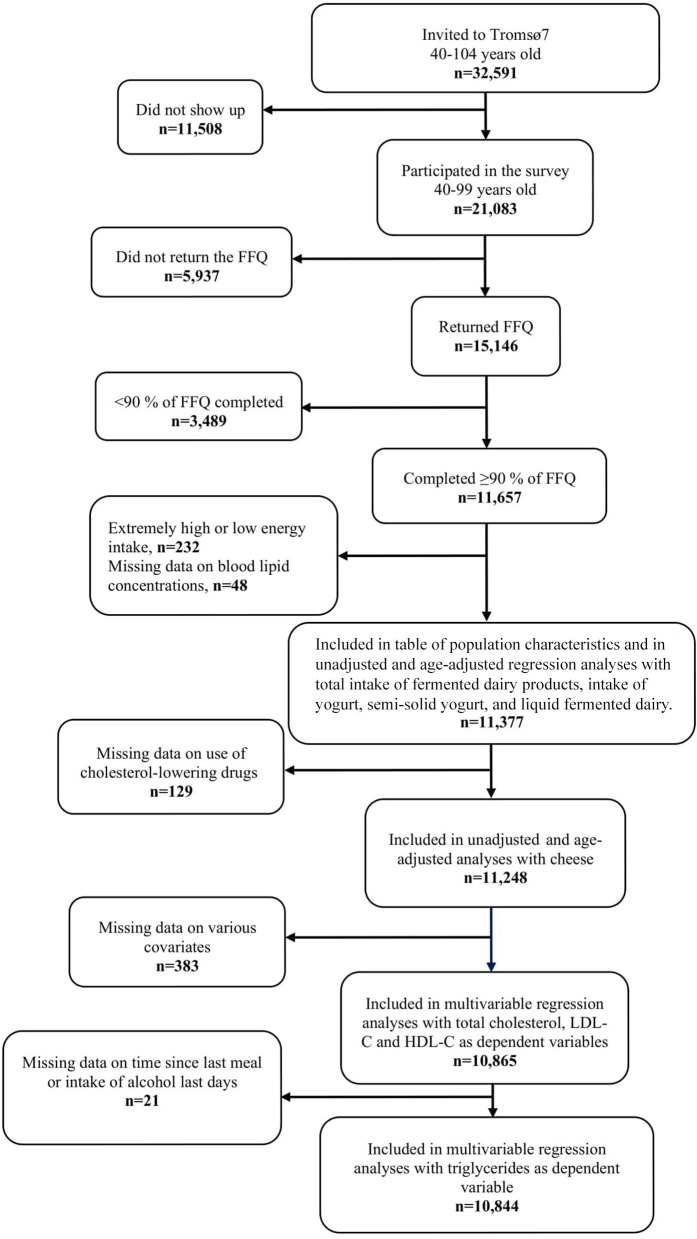
Flow chart of the study population. FFQ, food frequency questionnaire; LDL-C, low-density lipoprotein cholesterol; HDL-C, high-density lipoprotein cholesterol.

In Tromsø7, all individuals aged 40 years and older living in the Tromsø municipality were invited (*N* = 32,591), of which 21,083 men and women of 40–99 years of age attended (65 %) (24). In total 15,146 participants completed the FFQ, which is 72 % of those participating in Tromsø7 and 46 % of those originally invited. Participants that completed less than 90 % of the FFQ (*n* = 3,489), and those with the 1 % highest (above 21,267 kJ/day) or the 1 % lowest (below 3,948 kJ/day) total energy intake (*n* = 232) were excluded from the analyses, in accordance with previous cut-offs (26). In addition, participants with missing data on blood lipid concentrations (*n* = 48) were excluded, resulting in 11,377 participants eligible for the unadjusted and age-adjusted regression analyses with total intake of fermented dairy products, intake of liquid fermented dairy, and yogurt (including regular-fat and low-fat yogurt, and semi-solid yogurt) as exposure variables. The characteristics of these 11,377 participants are shown in Table 1. For the unadjusted and age-adjusted regression analyses with cheese intake (including regular-fat and low-fat cheese) as exposure variable (where the analyses were stratified by use of cholesterol-lowering drugs), an additional 129 participants were excluded because of missing data on use of cholesterol-lowering drugs, resulting in 11,248 participants (9,578 non-users and 1,670 users). In all the fully adjusted models, participants were excluded if they missed data on any of the included covariates. The fully adjusted models with total cholesterol, LDL-C, and HDL-C as dependent variables thus included 10,865 participants. In the regression analyses with triglycerides as dependent variable, an additional 21 participants were excluded due to missing data on information about time since last meal or missing data on intake of alcohol last two days, resulting in 10,844 participants eligible for these analyses. The flow chart of the study population is shown in Figure 1.

**Table 1:** Characteristics of the study population. The Tromsø Study: Tromsø7.

	**Total**	**Women**	**Men**
	**(*n =* 11,377)**	**(*n =* 6,074) (53.4 %)**	**(*n =* 5,303) (46.6 %)**
**Age, years**			
40–49	3,256 (29 %)	1,827 (30 %)	1,429 (27 %)
50–59	3,261 (29 %)	1,805 (30 %)	1,456 (27 %)
60–69	3,152 (28 %)	1,637 (27 %)	1,515 (29 %)
70+	1,708 (15 %)	805 (13 %)	903 (17 %)
**Education**			
Primary	2,314 (21 %)	1,263 (21 %)	1,051 (20 %)
Upper secondary	3,051 (27 %)	1,532 (25 %)	1,519 (29 %)
Tertiary short <4 years	2,316 (21 %)	1,110 (18 %)	1,206 (23 %)
Tertiary long ≥4 years	3,574 (32 %)	2,109 (35 %)	1,465 (28 %)
**Body mass index, kg/m** ^**2**^			
<25	3,770 (33 %)	2,422 (40 %)	1,348 (25 %)
25–29.9	4,982 (44 %)	2,300 (38 %)	2,682 (51 %)
30+	2,598 (23 %)	1,335 (22 %)	1,263 (24 %)
**Physical activity level**			
Sedentary	1,447 (13 %)	747 (13 %)	700 (13 %)
Light	6,531 (59 %)	3,840 (65 %)	2,691 (52 %)
Moderate	2,805 (25 %)	1,185 (20 %)	1,620 (31 %)
Vigorous	333 (3 %)	135 (2 %)	198 (4 %)
Smoking	1,422 (13 %)	816 (14 %)	606 (11 %)
Use of cholesterol-lowering drugs	1,670 (15 %)	732 (12 %)	938 (18 %)
Energy intake, kJ/day	9,722 ± 3,049	8,910 ± 2,739	10,652 ± 3,120
Fiber, g/day	28 ± 10	28 ± 10	28 ± 10
Polyunsaturated fat, E%	6.0 ± 1.6	6.0 ± 1.5	6.1 ± 1.6
Sugar, E%^a^	5.6 ± 3.5	5.4 ± 3.5	5.7 ± 3.6
Fermented dairy intake, g/day	131 (44–291)	146 (56–300)	113 (33–274)
Fermented dairy intake, g/10 MJ	140 (49–320)	171 (68–348)	104 (34–273)
Fermented liquid dairy intake, g/day	0 (0–200)	0 (0–200)	0 (0–200)
Yogurt, g/day	26 (4–112)	45 (9–118)	17 (0–90)
Yogurt, g/10 MJ	29 (4–112)	45 (10–139)	16 (0–80)
Low-fat yogurt, g/day	0 (0–57)	0 (0–100)	0 (0–9)
Regular-fat yogurt, g/day	11 (0–40)	15 (0–45)	9 (0–28)
Cheese, g/day	19 (10–36)	22 (13–40)	16 (7–29)
Cheese, g/10 MJ	21 (10–38)	26 (14–46)	16 (8–29)
Low-fat cheese, g/day	0 (0–4)	0 (0–7)	0 (0–0)
Regular-fat cheese, g/day	14 (7–29)	16 (7–29)	13 (7–29)
Total cholesterol, mmol/L	5.48 ± 1.07	5.58 ± 1.07	5.37 ± 1.07
-Fasting at blood sampling (*n =* 861)	5.67 ± 1.11	5.74 ± 1.12	5.62 ± 1.10
-Users of cholesterol-lowering drugs (*n =* 1,670)	4.68 ± 0.99	4.96 ± 0.98	4.46 ± 0.94
LDL-C, mmol/L	3.59 ± 0.99	3.58 ± 0.98	3.60 ± 1.00
-Fasting at blood sampling (*n =* 861)	3.82 ± 1.05	3.77 ± 1.06	3.87 ± 1.04
-Users of cholesterol-lowering drugs (*n =* 1,670)	2.82 ± 0.86	2.95 ± 0.87	2.72 ± 0.85
HDL-C, mmol/L	1.59 ± 0.49	1.75 ± 0.50	1.41 ± 0.41
-Fasting at blood sampling (*n =* 861)	1.55 ± 0.47	1.74 ± 0.47	1.39 ± 0.41
-Users of cholesterol-lowering drugs (*n =* 1,670)	1.51 ± 0.48	1.70 ± 0.51	1.36 ± 0.40
Triglycerides, mmol/L	1.30 (0.90–1.80)	1.13 (0.80–1.60)	1.43 (1.00–2.09)
-Fasting at blood sampling (*n =* 861)	1.18 (0.80–1.70)	1.00 (0.79–1.40)	1.33 (1.00–1.90)
-Users of cholesterol-lowering drugs (*n =* 1,670)	1.40 (1.00–2.00)	1.30 (0.92–1.80)	1.46 (1.10–2.01)

*Data is presented as frequency (percentage), mean (± standard deviation) or median (25th-75th percentile), where appropriate*.

a *Added sugar*.

### Dietary Data

Data on habitual dietary intake was collected through a 13-page FFQ, which was handed out to the participants at the examination site and completed at site or returned by mail. The FFQ contained questions about frequency and amount of 261 different types of food, dishes, dietary supplements, meals, and beverages, including alcoholic beverages, during the last year. The FFQ was developed at the University of Oslo (UiO) and is validated for estimating average energy and nutrient intake, however, not specifically for fermented dairy intake (27). Daily intake of total energy in kilojoule (kJ/day), food in grams (g/day), and nutrients in energy percentage (E%) and g/day were calculated using the food database KBS AE14 and KBS software system at UiO (KBS, version 7.3.). Details of the FFQ and the estimation of dietary intake is presented in Lundblad et al. (26).

### Intake of Fermented Dairy Products

Intake of fermented dairy products was estimated by the questions provided by the FFQ. The participants estimated the average frequencies and amounts of intake as never/seldom, or from ½ up to 7+ glasses (1 glass = 2 dl)/day for the following categories of liquid dairy products: whole milk/cultured milk/kefir, skimmed milk/skimmed cultured milk, milk with probiotics, and yogurt drinks. The intake of different yogurt products was categorized as ½, 1–3+ cups (125 g/169 g) in the following frequency categories: never/seldom, 1, 2, 3 times/month, or 1–8+ times/week. Cheese intake was categorized as number of slices of bread with semi-hard/hard cheese (including low-fat alternatives), dessert cheese, cheese spread (including low-fat alternatives) and cottage cheese in the following frequency categories: never/seldom, or 1–7, 8–12, 13–18, 19–24, 25–30, 31+ slices of bread with cheese/week. Intake of sour cream (10-, 20- and 35 % fat) was categorized as ½, 1, 1.5, 2, 3+ tablespoons in the following frequency categories: never/seldom, or 1–9+ times/month. The fermented dairy products were categorized into three main groups: Yogurt (both plain and sweetened alternatives: milk with probiotics, yogurt drinks, and other yogurt products), cheese [semi-hard/hard white cheese (including low-fat alternatives), dessert cheese, cheese spread (including low-fat alternatives), and cottage cheese], and total intake of fermented dairy products [yogurt (different types), cheese (different types), sour cream, and whole milk/cultured milk/kefir]. The low-fat yogurt category was defined as yogurt with <3 % fat, and included milk with probiotics, yogurt drinks, and low-fat yogurt products. The low-fat cheese category consisted of low-fat alternative cheese products and cottage cheese. The semi-solid yogurt category consisted of all yogurt products, except for milk with probiotics and yogurt drinks, as these were considered as liquid fermented dairy.

### Blood Sampling

Non-fasting venous blood samples were collected at the examination site with the participant sitting, with standard methods by trained technicians using a brief venous stasis applied to the upper arm, which was released before venipuncture. The blood samples were analyzed for serum total cholesterol, LDL-C, HDL-C, and triglycerides by enzymatic colorimetric methods with a Cobas 8000 c702 (Roche Diagnostics, Mannheim, Germany) at the Department of Laboratory Medicine, University Hospital of North Norway (ISO certificate NS-EN ISO 1-5189).

### Anthropometric Measurements

Height and weight were measured with an automatic electronic stadiometer [Jenix^®^ height & weight scale DS-103 (Jenix Co, Ltd)]. Body mass index (BMI) was calculated by dividing the weight in kilograms (kg) with the square root of the height in meters (m).

### Covariate Categories From Questionnaires

Information about educational level, smoking status, and use of cholesterol-lowering drugs was provided from the questionnaires based on the questions “What is the highest levels of education you have completed?” (primary, upper secondary, tertiary short [<4 years], and tertiary long [≥4 years]), “Do you/did you smoke daily” (Never/yes, now/yes, previously), and “Do you use, or have you used cholesterol lowering drugs?” (Never used/currently/previously, not now). The smoking status variable was dichotomized into current smoker vs. never or previously smoker, and the use of cholesterol-lowering drugs variable was dichotomized into current user of cholesterol-lowering drugs vs. never or previously user. Leisure-time physical activity level was based on the Saltin and Grimby questionnaire (28). The participants categorized their average exercise and physical exertion in leisure time over the last year as one of four mutually exclusive categories: “Reading, watching TV, or other sedentary activity” (sedentary); “Walking, cycling, or other forms of exercise at least four h a week (with examples)” (light physical activity level); “Participation in recreational sports, heavy gardening, etc. at least four h a week” (moderate physical activity level); or “Participation in hard training or sports competitions, regularly several times a week” (vigorous physical activity level). At blood sampling, the participants were asked about time since last meal (<1 h, 1–1.59, 2–2.59, 3–3.59, 4–4.59, 5–5.59, 6–6.59, 7–7.59, 8–8.59, 9+ h) and when they last consumed alcohol (0–2 days ago, 3–6 days ago, 7+ days ago, do not drink alcohol). If time since last meal was more than seven hours ago, it was defined as fasting and categorized accordingly, resulting in the following categories for the regression analyses: (<1 h, 1–1.59, 2–2.59, 3–3.59, 4–4.59, 5–5.59, 6–6.59, 7+ h). The participants' last consumption of alcohol was dichotomized into consumption 0–2 days ago (yes/no).

### Ethics

Data collection for Tromsø7 is approved by the Regional Committees for Medical and Health Research Ethics (REC north ref. 2014/940), and all participants gave their written informed consent.

### Statistical Analyses

Statistical analyses were performed with the statistical program IBM SPSS for Windows, version 26 (IBM Corp., Armonk, N.Y., USA). A two-sided *p-*value < 0.05 was considered as statistically significant.

Linear regression was used to investigate the associations between serum concentrations of total cholesterol, LDL-C, HDL-C, and triglycerides as dependent variables, and self-reported total intake of fermented dairy products, intake of liquid fermented dairy products, intake of yogurt (including low-fat yogurt, regular-fat yogurt and semi-solid yogurt), and intake of cheese (including low-fat and regular-fat cheese) as continuous exposure variables in three different models: an unadjusted model, an age-adjusted model, and a multivariable model. Other independent variables in the analyses were possible confounders, chosen based on preexisting literature and knowledge. The multivariable models included the following covariates: age (categorized into 10-year intervals: 40–49 years, 50–59 years, 60–69 year, and 70 years or older), sex, education (as described above), physical activity (as described above), BMI (categorized into standard groups: <25, 25–29.9, 30+ kg/m^2)^, energy intake (kJ/day), intake of polyunsaturated fat (E%), fiber (g/day), and alcohol (E%), use of cholesterol-lowering drugs (yes/no), and smoking (yes/no). In addition, the multivariable models that included triglycerides as dependent variable were adjusted for intake of sugar (E%), time since last meal (as described above), and intake of alcohol last two days (yes/no). Triglycerides were log10 transformed prior to the analyses, and the resulting regression coefficients were back transformed (exponentiating by 10). To show relative change per unit increase in the exposure, this number was subtracted with 1, and multiplied by 100. In the regression analyses, the units for the fermented dairy intake variables were chosen based on intake levels corresponding to approximately the interquartile range (IQR); 250 g/day for total intake of fermented dairy, 100 g/day for yogurt, and 25 g/day for cheese (calculated from Table 1). Interactions between the main groups of fermented dairy intake (total intake of fermented dairy, total intake of yogurt, and total intake of cheese) and use of cholesterol-lowering drugs, sex, and BMI were assessed one at a time by including cross-product terms with dairy intake and each variable in the multivariable models. As there was an interaction between intake of cheese and use of cholesterol-lowering drugs for all blood lipids, the analyses with cheese as exposure were performed stratified by use of cholesterol-lowering drugs (yes/no).

Model assumptions were assessed by graphical inspection of residuals, the normality assumption by histograms, and the homoscedasticity by scatter plots of residual vs. predicted values.

## Results

### Study Population Characteristics

Characteristics of the study population are shown in Table 1. The proportion of women was 53.4%, and 15% of the study population were using cholesterol-lowering drugs. The use of cholesterol-lowering drugs was more common among men (18%) than among women (12%). Median total intake of fermented dairy products was 131 g/day (146 g/day in women and 113 g/day in men), median intake of yogurt was 26 g/day (45 g/day in women and 17 g/day in men), and median intake of cheese was 19 g/day (22 g/day in women and 16 g/day in men). Mean concentration of total cholesterol, LDL-C, and HDL-C was 5.48 mmol/l, 3.59 mmol/l, and 1.59 mmol/l, respectively, while the median triglyceride concentration was 1.30 mmol/l. Women had higher concentration of total cholesterol and HDL-C, and lower concentration of triglycerides, compared to men.

### Associations Between Intake of Different Fermented Dairy Products and Blood Lipid Concentrations

Associations between total intake of fermented dairy products and yogurt, and blood lipid concentrations are shown in Table 2. There was an inverse association between total intake of fermented dairy products and triglyceride concentrations in the fully adjusted model (relative change −1.11 % (95 % CI −1.96 %, −0.24 %) per 250 g/day increase in total intake of fermented dairy products). Total intake of fermented dairy was not associated with total cholesterol, LDL-C, or HDL-C in any of the fully adjusted models.

**Table 2:** Linear regression coefficients for the associations between blood lipids and total intake of fermented dairy products and intake of yogurt. The Tromsø Study: Tromsø7.

	**Total intake of fermented dairy (250 g/day)**			**Intake of yogurt (100 g/day)**		
	**β^a^**	**95 % CI**	***p*** **-value**	**β^a^**	**95 % CI**	***p*** **-value**
**Total cholesterol (mmol/L)**						
Unadjusted model^b^	−0.0075	(−0.0260, 0.0110)	0.43	0.0079	(−0.0054, 0.0212)	0.25
Age-adjusted model^b^	−0.0107	(−0.0289, 0.0075)	0.25	0.0043	(−0.0089, 0.0174)	0.52
Multivariable model^c^	−0.0054	(−0.0238, 0.0131)	0.57	0.0028	(−0.0104, 0.0159)	0.68
**LDL cholesterol (mmol/L)**						
Unadjusted model^b^	−0.0140	(−0.0311, 0.0030)	0.11	−0.0020	(−0.0143, 0.0104)	0.75
Age-adjusted model^b^	−0.0144	(−0.0313, 0.0026)	0.10	−0.0040	(−0.0162, 0.0082)	0.52
Multivariable model^c^	−0.0118	(−0.0289, 0.0053)	0.18	0.0004	(−0.0118, 0.0126)	0.95
**HDL cholesterol (mmol/L)**						
Unadjusted model^b^	0.0081	(−0.0004, 0.0165)	0.06	0.0137	(0.0076, 0.0199)	<0.001
Age-adjusted model^b^	0.0050	(−0.0035, 0.0134)	0.25	0.0121	(0.0060, 0.0182)	<0.001
Multivariable model^c^	0.0075	(−0.0003, 0.0153)	0.06	0.0020	(−0.0035, 0.0076)	0.47
**Triglycerides (% change)**						
Unadjusted model^b^	−0.74	(−1.62, 0.14)	0.10	−0.57	(−1.19, 0.07)	0.08
Age–adjusted model^b^	−0.79	(−1.66, 0.09)	0.08	−0.62	(−1.26, 0.004)	0.05
Multivariable model^d^	−1.11^e^	(−1.96, −0.24)	0.01	0.05	(−0.57, 0.67)	0.87

a *β (regression coefficient) represents mean change in blood lipid concentrations per one unit (250 g/day and 100 g/day) increase in total intake of fermented dairy products and intake of yogurt. For the models with triglycerides as dependent variable, the regression coefficient represents relative change (%) in triglyceride concentration for one unit increase (250 g/day and 100 g/day) in total intake of fermented dairy and intake of yogurt*.

b *n = 11,377*.

c *Adjusted for age, sex, education, level of physical activity, body mass index, energy intake, intake of polyunsaturated fatty acids, fiber, and alcohol, use of cholesterol-lowering drugs, and smoking status. n = 10,865*.

d *Adjusted for age, sex, education, level of physical activity, body mass intake, energy intake, intake of polyunsaturated fatty acids, fiber, sugar, and alcohol, use of cholesterol-lowering drugs, smoking status, time since last meal, and intake of alcohol last two days. n = 10,844*.

e *A 250 g/day increase in total intake of fermented dairy products is associated with a decrease of −0.01 mmol/L for a person that has a triglyceride concentration of 1.30 mmol/L (median value of the study population)*.

A positive association was seen between intake of yogurt and HDL-C in the unadjusted and age-adjusted models (regression coefficient 0.01 mmol/l (95 % CI 0.01, 0.02) and 0.01 mmol/l (95 % CI 0.01, 0.02), respectively, per 100 g/day increase in yogurt intake). However, the association did not remain significant in the fully adjusted model (*p* = 0.47). There was no association between intake of yogurt and total cholesterol, LDL-C, or triglycerides in any of the fully adjusted models (Table 2). When yogurt was analyzed based on fat content, neither low-fat yogurt nor regular-fat yogurt was associated with any of the blood lipids in the fully adjusted models (Table 3).

**Table 3:** Linear regression coefficients for the associations between blood lipids and intake of low-fat yogurt and intake of regular-fat yogurt. The Tromsø Study: Tromsø7.

	**Intake of low-fat yogurt (100 g/day)**			**Intake of regular-fat yogurt (100 g/day)**		
	**β^a^**	**95 % CI**	***p-*** **value**	**β^a^**	**95 % CI**	***p*** **-value**
**Total cholesterol (mmol/L)**						
Unadjusted model^b^	0.0074	(−0.0068, 0.0215)	0.31	0.0821	(0.0589, 0.1052)	<0.001
Age–adjusted model^b^	0.0039	(−0.0101, 0.0178)	0.56	0.0118	(−0.0380, 0.0615)	0.64
Multivariable model^c^	0.0042	(−0.0095, 0.0179)	0.55	−0.0143	(−0.0630, 0.0344)	0.56
**LDL cholesterol (mmol/L)**						
Unadjusted model^b^	−0.0007	(−0.0138, 0.0124)	0.92	−0.0199	(−0.0666, 0.0268)	0.40
Age-adjusted model^b^	−0.0024	(−0.0154, 0.0105)	0.71	−0.0260	(−0.0722, 0.0202)	0.27
Multivariable model^c^	0.0020	(−0.0107, 0.0147)	0.76	−0.0201	(−0.0652, 0.0250)	0.38
**HDL cholesterol (mmol/L)**						
Unadjusted model^b^	0.0090	(0.0025, 0.0156)	0.01	0.0821	(0.0589, 0.1052)	<0.001
Age-adjusted model^b^	0.0073	(0.0008, 0.0138)	0.03	0.0808	(0.0578, 0.1038)	<0.001
Multivariable model^c^	0.0010	(−0.0048, 0.0068)	0.74	0.0154	(−0.0051, 0.0360)	0.14
**Triglycerides (% change)**						
Unadjusted model^b^	−0.06	(−0.73, 0.62)	0.87	−7.14	(−9.36, −4.87)	<0.001
Age-adjusted model^b^	−0.12	(−0.79, 0.56)	0.73	−7.30	(−9.51, −5.04)	<0.001
Multivariable model^d^	0.23	(−0.41, 0.89)	0.48	−2.22	(−4.45, 0.05)	0.06

a *β (regression coefficient) represents mean change in blood lipid concentrations per one unit (100 g/day) increase in intake of low-fat yogurt and intake of regular-fat yogurt. For the models with triglycerides as dependent variable, the regression coefficient represents relative change (%) in triglyceride concentration for one unit increase (100 g/day) in intake of low-fat yogurt and regular-fat yogurt*.

b *n = 11,377*.

c *Adjusted for age, sex, education, level of physical activity, body mass index, energy intake, intake of polyunsaturated fatty acids, fiber, and alcohol, use of cholesterol-lowering drugs, and smoking status. n = 10,865*.

d *Adjusted for age, sex, education, level of physical activity, body mass intake, energy intake, intake of polyunsaturated fatty acids, fiber, sugar, and alcohol, use of cholesterol-lowering drugs, smoking status, time since last meal, and intake of alcohol last two days. n = 10,844*.

The associations between semi-solid yogurt and liquid fermented dairy, and blood lipid concentrations are shown in Table 4. Intake of semi-solid yogurt was inversely associated with LDL-C and triglycerides (regression coefficient−0.04 mmol/l (95 % CI−0.08, −0.0001) and relative change −2.48 % (95 % CI −4.38 %, −0.55 %), respectively, per 100 g/day increase in semi-solid yogurt intake). There were no associations between liquid fermented dairy intake and blood lipid concentrations.

**Table 4:** Linear regression coefficients for the associations between blood lipids and intake of semi-solid yogurt and intake of liquid fermented dairy. The Tromsø Study: Tromsø7.

	**Intake of semi-solid yogurt (100 g/day)**			**Intake of liquid fermented dairy (100 g/day)**		
	**β^a^**	**95 % CI**	***p-*** **value**	**β^a^**	**95 % CI**	***p*** **-value**
**Total cholesterol (mmol/L)**						
Unadjusted model^b^	−0.0070	(−0.0497, 0.0357)	0.75	−0.0029	(−0.0106, 0.0047)	0.45
Age-adjusted model^b^	−0.0191	(−0.0612, 0.0230)	0.37	−0.0040	(−0.0115, 0.0035)	0.30
Multivariable model^c^	−0.0375	(−0.0790, 0.0040)	0.08	−0.0007	(−0.0082, 0.0068)	0.86
**LDL cholesterol (mmol/L)**						
Unadjusted model^b^	−0.0451	(−0.0846, −0.0056)	0.03	−0.0036	(−0.0107, 0.0035)	0.32
Age–adjusted model^b^	−0.0563	(−0.0953, −0.0172)	0.01	−0.0033	(−0.0103, 0.0037)	0.35
Multivariable model^c^	−0.0385	(−0.0770, −0.0001)	0.049	−0.0029	(−0.0098, 0.0040)	0.41
**HDL cholesterol (mmol/L)**						
Unadjusted model^b^	0.0765	(0.0569, 0.0960)	<0.001	−0.0006	(−0.0041, 0.0029)	0.73
Age-adjusted model^b^	0.0765	(0.0571, 0.0960)	<0.001	−0.0021	(−0.0056, 0.0014)	0.23
Multivariable model^c^	0.0098	(−0.0077, 0.0273)	0.27	0.0021	(−0.0010, 0.0053)	0.19
**Triglycerides (% change)**						
Unadjusted model^b^	−6.87	(−8.75, −4.94)	<0.001	0.04	(−0.32, 0.41)	0.82
Age–adjusted model^b^	−7.12	(−8.99, −5.20)	<0.001	0.03	(−0.33, 0.40)	0.86
Multivariable model^d^	−2.48^e^	(−4.38, −0.55)	0.01	−0.32	(−0.68, 0.03)	0.07

a *β (regression coefficient) represents mean change in blood lipid concentrations per one unit (100 g/day) increase in intake of semi-solid yogurt and intake of liquid fermented dairy. For the models with triglycerides as dependent variable, the regression coefficient represents relative change (%) in triglyceride concentration for one unit increase (100 g/day) in intake of semi-solid yogurt and intake of liquid fermented dairy*.

b *n = 11,377*.

c *Adjusted for age, sex, education, level of physical activity, body mass index, energy intake, intake of polyunsaturated fatty acids, fiber, and alcohol, use of cholesterol-lowering drugs, and smoking status. n = 10,865*.

d *Adjusted for age, sex, education, level of physical activity, body mass intake, energy intake, intake of polyunsaturated fatty acids, fiber, sugar, and alcohol, use of cholesterol-lowering drugs, smoking status, time since last meal, and intake of alcohol last two days. n = 10,844. e. A 100 g/day increase in intake of semi-solid yogurt is associated with a decrease of −0.03 mmol/L for a person that has a triglyceride concentration of 1.30 mmol/L (median value of the study population)*.

The associations between cheese intake and blood lipid concentrations varied by use of cholesterol-lowering drugs (see Table 5). For non-users, cheese intake was inversely associated with LDL-C and triglycerides, and positively associated with HDL-C, with regression coefficients per 25 g/day increase in cheese intake from the fully adjusted models being −0.03 mmol/l (95 % CI −0.04, −0.01), −1.34% (95 % CI −2.29%, −0.37%), and 0.02 mmol/l (95 % CI 0.01, 0.03), respectively. There was an inverse association between cheese intake and total cholesterol in the unadjusted and age-adjusted models, but not in the fully adjusted model (regression coefficient −0.02 mmol/l (95 % CI −0.04, 0.01) per 25 g/day increase in cheese intake). For users of cholesterol-lowering drugs, there were no associations between intake of cheese and any of the blood lipids, but the regression coefficients pointed toward opposite associations compared to the non-users.

**Table 5:** Linear regression coefficients for the associations between blood lipids and intake of cheese. The Tromsø Study: Tromsø7.

	**Intake of cheese (25 g/day)**					
	**Non-users of cholesterol-lowering drugs (*n* = 9,578)**			**Users of cholesterol-lowering drugs (*n* = 1,670)**		
	**β^a^**	**95 % CI**	***p*-value**	**β^a^**	**95 % CI**	***p*-value**
**Total cholesterol (mmol/L)**						
Unadjusted model	−0.0342	(−0.0540, −0.0144)	0.001	0.0443	(−0.0084, 0.0970)	0.10
Age-adjusted model	−0.0211	(−0.0403, −0.0020)	0.03	0.0392	(−0.0132, 0.0916)	0.14
Multivariable model^b^	−0.0150	(−0.0352, 0.0053)	0.15	0.0377	(−0.0174, 0.0927)	0.18
**LDL cholesterol (mmol/L)**						
Unadjusted model	−0.0548	(−0.0732, −0.0364)	<0.001	0.0372	(−0.0090, 0.0833)	0.11
Age-adjusted model	−0.0460	(−0.0641, −0.0280)	<0.001	0.0330	(−0.0128, 0.0787)	0.16
Multivariable model^b^	−0.0250	(−0.0438, −0.0061)	0.01	0.0357	(−0.0144, 0.0857)	0.16
**HDL cholesterol (mmol/L)**						
Unadjusted model	0.0385	(0.0290, 0.0480)	<0.001	0.0025	(−0.0234, 0.0284)	0.85
Age-adjusted model	0.0433	(0.0339, 0.0527)	<0.001	0.0031	(−0.0226, 0.0288)	0.81
Multivariable model^b^	0.0178	(0.0093, 0.0264)	<0.001	−0.0158	(−0.0403, 0.0087)	0.21
**Triglycerides (% change)**						
Unadjusted model	−3.23	(−4.20, −2.26)	<0.001	−0.26	(−2.86, 2.40)	0.85
Age-adjusted model	−3.23	(−4.19, −2.26)	<0.001	−0.61	(−3.16, 2.01)	0.65
Multivariable model^c^	−1.34^d^	(−2.29, −0.37)	0.01	1.23	(−1.46, 4.00)	0.37

a *β (regression coefficient) represents mean change in blood lipid concentrations per one unit (25 g/day) increase in intake of cheese. For the models with triglycerides as dependent variable, the regression coefficient represents relative change (%) in triglyceride concentrations for one unit (25 g/day) increase in intake of cheese*.

b *Adjusted for age, sex, education, level of physical activity, body mass index, energy intake, intake of polyunsaturated fatty acids, fiber, and alcohol, and smoking status. n = 9,303 for non-users of cholesterol-lowering drugs and n = 1,562 for users of cholesterol-lowering drugs*.

c *Adjusted for age, sex, education, level of physical activity, body mass intake, energy intake, intake of polyunsaturated fatty acids, fiber, sugar, and alcohol, smoking status, time since last meal, and intake of alcohol last two days. n = 9,285 for non-users of cholesterol-lowering drugs and n = 1,559 for users of cholesterol-lowering drugs*.

d *A 25 g/day increase in intake of cheese is associated with a decrease of −0.02 mmol/L for a person that has triglyceride concentration of 1.30 mmol/L (median value of the study population)*.

Low-fat cheese and regular-fat cheese were differently associated with the blood lipids, as low-fat cheese was inversely associated with total cholesterol, LDL-C, and triglycerides among non-users of cholesterol-lowering drugs only (regression coefficients −0.05 mmol/l (95 % CI −0.09, −0.01), −0.05 mmol/l (95 % CI −0.08, −0.01) and relative change −3.87% (95 % CI −5.56% −2.16%), respectively, per 25 g/day increase in low-fat cheese intake) (Table 6). Intake of regular-fat cheese was positively associated with HDL-C among non-users of cholesterol-lowering drugs [regression coefficient 0.02 mmol/l (95 % CI 0.01, 0.03)] (Table 6) and positively associated with total cholesterol among users of cholesterol-lowering drugs [regression coefficient 0.07 mmol/l (95 % CI 0.002, 0.13)] (Table 7) per 25 g/day increase in regular-fat cheese intake.

**Table 6:** Linear regression coefficients for the associations between blood lipids and intake of low-fat cheese and intake of regular-fat cheese among non-users of cholesterol-lowering drugs (*n* = 9,578). The Tromsø Study: Tromsø7.

	**Non-users of cholesterol-lowering drugs (*n* = 9,578)**					
	**Intake of low-fat cheese (25 g/day)**			**Intake of regular-fat cheese (25 g/day)**		
	**β^a^**	**95 % CI**	***p*** **-value**	**β^a^**	**95 % CI**	***p*** **-value**
**Total cholesterol (mmol/L)**						
Unadjusted model^b^	−0.0493	(−0.0867, −0.0119)	0.01	−0.0279	(−0.0511, −0.0047)	0.02
Age-adjusted model^b^	−0.0476	(−0.0837, −0.0115)	0.01	−0.0105	(−0.0330, 0.0119)	0.36
Multivariable model^c^	−0.0502	(−0.0873, −0.0130)	0.01	−0.0001	(−0.0236, 0.0235)	0.997
**LDL cholesterol (mmol/L)**						
Unadjusted model^b^	−0.0779	(−0.1127, −0.0432)	<0.001	−0.0450	(−0.0666, −0.0235)	<0.001
Age-adjusted model^b^	−0.0776	(−0.1115, −0.0436)	<0.001	−0.0330	(−0.0541, −0.0119)	0.002
Multivariable model^c^	−0.0490	(−0.0835, −0.0144)	0.01	−0.0140	(−0.0360, 0.0079)	0.21
**HDL cholesterol (mmol/L)**						
Unadjusted model^b^	0.0658	(0.0479, 0.0838)	<0.001	0.0274	(0.0162, 0.0385)	<0.001
Age-adjusted model^b^	0.0678	(0.0500, 0.0855)	<0.001	0.0331	(0.0220, 0.0441)	<0.001
Multivariable model^c^	0.0137	(−0.0020, 0.0294)	0.09	0.0186	(0.0087, 0.0285)	<0.001
**Triglycerides (% change)**						
Unadjusted model^b^	−7.77	(−9.49, −6.02)	<0.001	−1.37	(−2.52, −0.21)	0.02
Age-adjusted model^b^	−7.87	(−9.58, −6.12)	<0.001	−1.32	(−2.47, −0.15)	0.03
Multivariable model^d^	−3.87^e^	(−5.56, −2.16)	<0.001	−0.21	(−1.32, 0.92)	0.72

a *β (regression coefficient) represents mean change in blood lipid concentrations per one unit (25 g/day) increase in intake of low-fat cheese and intake of regular-fat cheese. For the models with triglycerides as dependent variable, the regression coefficient represents relative change (%) in triglyceride concentration for one unit increase (25 g/day) in intake of low-fat cheese and intake of regular-fat cheese*.

b *n = 9,578*.

c *Adjusted for age, sex, education, level of physical activity, body mass index, energy intake, intake of polyunsaturated fatty acids, fiber, and alcohol, and smoking status. n = 9,303*.

d *Adjusted for age, sex, education, level of physical activity, body mass intake, energy intake, intake of polyunsaturated fatty acids, fiber, sugar, and alcohol, smoking status, time since last meal, and intake of alcohol last two days. n = 9,285*.

e *A 25 g/day increase in intake of low-fat cheese is associated with a decrease of −0.05 mmol/L for a person that has triglyceride concentration of 1.30 mmol/L (median value of the study population)*.

**Table 7:** Linear regression coefficients for the associations between blood lipids and intake of low-fat cheese and intake of regular-fat cheese among users of cholesterol-lowering drugs (*n* = 1,670). The Tromsø Study: Tromsø7.

	**Users of cholesterol-lowering drugs (*n* = 1,670)**					
	**Intake of low-fat cheese (25 g/day)**			**Intake of regular-fat cheese (25 g/day)**		
	**β^a^**	**95 % CI**	***p*** **-value**	**β^a^**	**95 % CI**	***p*** **-value**
**Total cholesterol (mmol/L)**						
Unadjusted model^b^	0.0287	(−0.0583, 0.1157)	0.52	0.0512	(−0.0138, 0.1161)	0.12
Age-adjusted model^b^	0.0152	(−0.0712, 0.1017)	0.73	0.0509	(−0.0136, 0.1154)	0.12
Multivariable model^c^	−0.0252	(−0.1145, 0.0642)	0.58	0.0684	(0.0022, 0.1346)	0.04
**LDL cholesterol (mmol/L)**						
Unadjusted modell^b^	0.0037	(−0.0724, 0.0799)	0.92	0.0543	(−0.0026, 0.1111)	0.06
Age-adjusted model^b^	−0.0090	(−0.0846, 0.0665)	0.82	0.0549	(−0.0014, 0.1113)	0.06
Multivariable model^c^	−0.0124	(−0.0937, 0.0689)	0.77	0.0585	(−0.0017, 0.1187)	0.06
**HDL cholesterol (mmol/L)**						
Unadjusted model^b^	0.0297	(−0.0130, 0.0723)	0.17	−0.0128	(−0.0447, 0.0191)	0.43
Age-adjusted model^b^	0.0345	(−0.0079, 0.0768)	0.11	−0.0145	(−0.0461, 0.0172)	0.37
Multivariable model^c^	−0.0257	(−0.0654, 0.0141)	0.21	−0.0088	(−0.0382, 0.0207)	0.56
**Triglycerides (% change)**						
Unadjusted model^b^	−0.46	(−4.69, 3.97)	0.84	−0.14	(−3.33, 3.15)	0.93
Age-adjusted model^b^	−1.34	(−5.48, 2.98)	0.54	−0.17	(−3.31, 3.07)	0.92
Multivariable model^d^	1.25	(−3.08, 5.76)	0.58	1.10	(−2.14, 4.45)	0.51

a *β (regression coefficient) represents mean change in blood lipid concentrations per one unit (25 g/day) increase in intake of low-fat cheese and intake of regular-fat cheese. For the models with triglycerides as dependent variable, the regression coefficient represents relative change (%) in triglyceride concentration for one unit increase (25 g/day) in intake of low-fat cheese and intake of regular-fat cheese*.

b *n = 1,670*.

c *Adjusted for age, sex, education, level of physical activity, body mass index, energy intake, intake of polyunsaturated fatty acids, fiber, and alcohol, and smoking status. n = 1,562*.

d *Adjusted for age, sex, education, level of physical activity, body mass intake, energy intake, intake of polyunsaturated fatty acids, fiber, sugar, and alcohol, smoking status, time since last meal, and intake of alcohol last two days. n = 1,559*.

## Discussion

In this cross-sectional study, main findings were that cheese intake was positively associated with HDL-C, and inversely associated with LDL-C and triglyceride concentrations among those not using cholesterol-lowering drugs, while total intake of fermented dairy products was inversely associated with triglyceride concentrations only, and yogurt not associated with any of the blood lipids. Both fat content and dairy matrix seemed to affect the associations with blood lipid concentrations, as low-fat cheese showed favorable associations compared to regular-fat cheese, and intake of semi-solid yogurt was inversely associated with LDL-C and triglycerides, while no associations were found for total yogurt intake or fermented liquid dairy intake.

There was a significant positive association between yogurt intake and HDL-C in the unadjusted and age-adjusted models, but not in the fully adjusted model, where several possible confounding variables were added. These included sex, age, educational level, physical activity level, BMI, energy intake, intake of polyunsaturated fatty acids, fiber, and alcohol, use of cholesterol-lowering drugs, and smoking status. This is in accordance with findings from a cross-sectional analysis of Australian elderly women by Ivey et al. (29) where yogurt intake was positively associated with HDL-C in the unadjusted and the age-adjusted model, but not in the multivariable model, indicating that the positive association could be due to lifestyle habits and dietary factors. Moreover, the same study didn't find an association between yogurt intake and LDL-C (29), as was also seen in this cross-sectional study.

Cheese intake was positively associated with HDL-C, and inversely associated with LDL-C and triglycerides among those not using cholesterol-lowering drugs, but no association was found for those using cholesterol-lowering drugs. The reason for the observed difference between users and non-users could be that the cholesterol-lowering drugs have such a strong impact on the blood lipids that differences due to cheese intake become hard to discover. Furthermore, one could expect those using cholesterol-lowering drugs to have higher cardiovascular risk, influencing the relation between cheese intake and blood lipid concentrations. There could also be differences in diets or other lifestyle factors between the groups, possibly as those using cholesterol-lowering drugs have had another dietary pattern and follow lifestyle or dietary advices. Interestingly, the associations between cheese intake and blood lipid concentrations among those using cholesterol-lowering drugs were opposite to those not using cholesterol-lowering drugs in the multivariable models, indicating potential adverse effects of cheese intake on blood lipids among those using cholesterol-lowering drugs. These associations were, however, not statistically significant.

Several cross-sectional studies have examined the associations between fermented dairy intake and blood lipid concentrations. The analyses by Ivey et al. on Australian women showed no associations between cheese intake and HDL-C or LDL-C (29). These analyses were not stratified by the use of cholesterol-lowering drugs, which we found to be an effect modifier, but were adjusted for use of vascular medication (which included HMG-CoA reductase inhibitors) (29). In a cross-sectional study of a Mediterranean population at high cardiovascular risk (30), the associations between total fermented dairy intake, as well as cheese and yogurt (including low-fat and whole-fat variants), and several components of the metabolic syndrome were examined. The results of the study were in accordance with the findings from this study, as cheese consumption was inversely associated with a low risk of low HDL-C plasma level and hypertriglyceridemia, while there was no significant association for total fermented dairy intake or yogurt intake (30). Further cross-sectional studies support a positive association between cheese intake and HDL-C (31–33), but there are conflicting results regarding cheese intake and LDL-C. There was no association between cheese intake and LDL-C in cross-sectional analyses of a Swedish population (32), while there was a positive association for men, but an inverse association for women, in a US population-based cross-sectional study, where participants taking cholesterol-lowering drugs were excluded from the analyses (31). The different association for men and women was suggested by the authors to may be explained by women selecting cheeses with lower saturated fat content compared to men. The study found no associations between intake of cheese and total cholesterol or triglycerides. The lack of an association between cheese intake and triglyceride concentration is supported by the Swedish study (32) and a previous Norwegian study (33), but is not in accordance with the observed inverse association in this study.

Even though cheese intake was favorably associated with blood lipid concentrations, the association seemed to be dependent on the fat content of the cheese, as low-fat cheese was inversely associated with total cholesterol, LDL-C, and triglycerides, while regular-fat cheese was not associated with these blood lipids, albeit being positively associated with HDL-C (Table 6). These findings only applied for participants not using cholesterol-lowering drugs, while for users of cholesterol-lowering drugs, there were no associations between intake of low-fat cheese and blood lipid concentrations. Regular-fat cheese was positively associated with total cholesterol concentrations in users of cholesterol-lowering drugs (Table 7). Possible explanations for the different associations between these two groups of people are discussed above. The associations found between low-fat cheese and blood lipids in this present study were statistically significant despite the intake of low-fat cheese in the study population being low, as only 32% of the population consumed low-fat cheese, with median intake among consumers being 11 g/day. In contrast to the fat content dependent associations between cheese intake and blood lipids, there were no significant associations between neither low-fat yogurt nor regular-fat yogurt and blood lipid concentrations (Table 3). A possible explanation for this could be that the fat content in yogurt is lower than in most cheese types, and the difference in fat content between the low-fat and regular-fat yogurt group is relatively small compared to the difference between the low-fat and regular-fat cheese group. The associations with HDL-C and triglycerides are in accordance with results from the cross-sectional study of a Mediterranean population at high cardiovascular risk mentioned above, as neither low-fat nor full-fat yogurt was significantly associated with risk of low HDL-C plasma level or hypertriglyceridemia (30). The results discussed in this section indicate that associations between cheese intake and blood lipid concentrations are affected by fat content and use of cholesterol-lowering drugs, which should be further investigated.

As cheese has a high content of saturated fat, the association found in this cross-sectional study between cheese intake and LDL-C among those not using cholesterol-lowering drugs is contradictive. On the other hand, a meta-analysis of RCTs found that consumption of hard cheese reduces LDL-C and HDL-C compared to butter with the same ratio of polyunsaturated fatty acids to saturated fatty acids, indicating that different dairy products could have different effects on blood lipids explained by other factors than the fatty acid composition (14). However, what causes the different effects remains to be elucidated. The reduction in HDL-C is not in accordance with the observed positive association in the present study, which potentially could be due to the lack of a comparison to butter. The monounsaturated fatty acid oleic acid (C18:1n-9 cis), which is the major unsaturated fatty acid in milk fat constituting 21% of the total fatty acid content in bovine milk (9), could potentially be part of an explanation of the contradictory association between cheese intake and blood lipids observed in the present study, as high intake of monounsaturated fatty acids may lower both plasma total cholesterol, LDL-C, and triglyceride concentrations (34). However, as this fatty acid is found in all dairy products, it is unlikely to explain the associations found for cheese specifically. The various effects on blood lipid concentrations from different dairy products may instead be related to the so-called dairy matrix. This concept proposes that the effects of dairy products should be considered as a function of the total nutrient content within the texture and structure of each specific dairy product, instead of being solely based on the content of single nutrients (35), as industrial processes can impact the nutrients and their interactions. Suggested modifiers of the expected blood lipid response from saturated fat are calcium, phosphorus, the milk fat globule membrane, and starter cultures (in fermented dairy products), presumably attenuating the expected blood lipid response by decreasing intestinal fat absorption and bile-acid recycling, modulating the gut microbiota, and/or altering gene expressions (35).

The different associations between blood lipid concentrations and total intake of yogurt, semi-solid yogurt, and liquid fermented dairy could potentially be explained by the dairy matrix concept described above, as various physical structures and textures could influence the health effects of different dairy foods (35). Intake of semi-solid yogurt was inversely associated with LDL-C and triglyceride concentrations, while there were no associations for total yogurt intake. As the only difference in dairy product intake between the two groups was that the total yogurt intake group included intake of low-fat yogurts, which were not included in the semi-solid yogurt group due to their liquid texture, it seems plausible that the different associations with LDL-C and triglycerides could be explained by differences in dairy matrix structures between the two groups. Supporting this explanation, intake of fermented liquid dairy products was not associated with blood lipid concentrations. However, the dairy matrix concept is complex, and which components and mechanisms that may have attributed to the observed associations remains to be determined.

The significant associations found between intake of fermented dairy products and blood lipids are modest. Per 1 mmol/l reduction in LDL-C, the relative risk of major vascular events decreases by about 23 % in people treated with statin therapy over 5 years (36–38). Thus, even if there is an actual effect on LDL-C by semi-solid yogurt intake (100 g/day) and cheese intake (total intake and low-fat cheese intake) (25 g/day) among non-users of cholesterol-lowering drugs, the observed 0.03–0.05 mmol/l decrease is likely of minor clinical relevance, when discussed as single food items.

### Strengths and Limitations

There are several strengths of this study. It is based on a large sample of 11,377 adult women and men from the general population, the analyses were adjusted for a wide range of confounding factors, and the data collection was performed by standard methods and procedures, including the use of a comprehensive and previously validated FFQ.

There are also some limitations of this study. Some of the questions in the FFQ concerning intake of the different dairy products are not optimal in the sense that both fermented and non-fermented dairy products are combined into one question, and likewise for yogurt and non-yogurt products. For example, whole milk, which is a non-fermented dairy product, is included in the same question as cultured milk and kefir, which are fermented dairy products. The design of these questions hinders the possibility to separate the different dairy products, which generates uncertainty to the results and potential information bias through measurement errors. Another limitation is that intakes of fermented dairy products consumed as part of dishes are lacking. This is likely to affect the estimate of cheese intake, but to a lesser extent the yogurt intake, since it can be assumed that cheese is more often used as part of different dishes. In addition, the intake of cheese was based on reported number of slices of bread with cheese, estimating the amount of cheese to be approximately 20 gram per slice of bread. Moreover, the FFQ does not include all the fermented dairy products available on the market, although including the most common ones.

Due to the observational design of this cross-sectional study, the associations between intake of the fermented dairy products and blood lipid concentrations do not imply causality. Also, intake of fermented dairy products, fermented milk, yogurt, and cheese has previously been shown to be related to a healthier lifestyle and healthier diet (30, 32), making it difficult to attribute separate effects to the fermented dairy products. In contrast to these studies, intake of cheese was associated with consumption of other foods high in saturated fat in a cross-sectional US population-based study (31), which suggests that dietary patterns related to cheese intake may vary between different food cultures. In our present study, the analyses were not adjusted for other foods high in saturated fat, and although adjusting for a wide range of potential confounding factors, we cannot be sure that all confounding factors were included, and that residual confounding can be ruled out. However, we adjusted for intake of known dietary modifiers of blood lipid concentrations, such as polyunsaturated fatty acids and fiber (39–41).

Also, it is important to highlight that the effect of different foods on health outcomes could be dependent on what the foods replace in the diet. For example, it has been shown that the association between dairy fat and risk of CVD is dependent of what dairy fat is being replaced with, as substitution of dairy fat with polyunsaturated fatty acid, vegetable fat or carbohydrates from whole grains is associated with a reduced risk of CVD, while substitution of dairy fat with refined starch and sugar is not associated with a different CVD risk (42). This means that associations or effects due to single food items should be interpreted cautiously, and with awareness of the possible impact and complexity of replacing different foods or nutrients in the diet.

In conclusion, this study found that the associations between intake of fermented dairy products and blood lipid concentrations were dependent on the type of dairy product, the fat content, and the dairy matrix structure. Among the fermented dairy products, cheese intake showed the most favorable associations being positively associated with HDL-C, and inversely associated with LDL-C and triglyceride concentrations among subjects not using cholesterol-lowering drugs. However, the associations between cheese and the blood lipids seemed to depend on the fat content, as low-fat cheese was more favorably associated with the blood lipids compared to regular-fat cheese. Regarding different dairy matrix structures, intake of semi-solid yogurt was inversely associated with LDL-C and triglycerides, while intake of liquid fermented dairy was not associated with any of the blood lipids. This study highlights the importance of investigating specific types of dairy products separately, based on fat content and dairy matrix, when examining effects on blood lipid concentrations, and stratifying statistical models by use of cholesterol-lowering drugs when relevant.

## Data Availability Statement

The data analyzed in this study is subject to the following licenses/restrictions: The dataset is available upon application to the Tromsø Study. Requests to access these datasets should be directed to www.uit.no/research/tromsostudy.

## Ethics Statement

The studies involving human participants were reviewed and approved by Regional Committees for Medical and Health Research Ethics (REC north). The patients/participants provided their written informed consent to participate in this study.

## Author Contributions

MM, LH, and PH designed the study. MM performed statistical analyses. TW provided statistical input. MM wrote the first draft of the manuscript. All authors contributed to manuscript revision, read, and approved the submitted version.

## Funding

UiT The Arctic University of Norway has signed an Open Access publishing agreement with Frontiers that includes pay-as-you-publish by the University library.

## Conflict of Interest

The authors declare that the research was conducted in the absence of any commercial or financial relationships that could be construed as a potential conflict of interest.

## Publisher's Note

All claims expressed in this article are solely those of the authors and do not necessarily represent those of their affiliated organizations, or those of the publisher, the editors and the reviewers. Any product that may be evaluated in this article, or claim that may be made by its manufacturer, is not guaranteed or endorsed by the publisher.
